# Fully resolved assembly of *Fusarium proliferatum* DSM106835 genome

**DOI:** 10.1038/s41597-023-02610-4

**Published:** 2023-10-16

**Authors:** Gouthaman P. Purayil, Amal Y. Almarzooqi, Khaled A. El-Tarabily, Frank M. You, Synan F. AbuQamar

**Affiliations:** 1 Department of Biology, College of Science, United Arab Emirates University, Al Ain, 15551 United Arab Emirates; 2 Ottawa Research and Development Centre, Agriculture and Agri-Food Canada, 960 Carling Avenue, Ottawa, ON K1A 0C6 Canada

**Keywords:** Microbe, Comparative genomics

## Abstract

In the United Arab Emirates, sudden decline syndrome (SDS) is a destructive disease of date palm caused by the soil-borne fungal pathogen *Fusarium proliferatum* (*Fp*) DSM106835. Here, a high-resolution genome assembly of *Fp* DSM106835 was generated using PacBio HiFi sequencing with Omni-C data to provide a high-quality chromatin-organised reference genome with 418 scaffolds, totalling 58,468,907 bp in length and an N50 value of 4,383,091 bp from which 15,580 genes and 16,321 transcripts were predicted. The assembly achieved a complete BUSCO score of 99.2% for 758 orthologous genes. Compared to seven other *Fp* strains, *Fp* DSM106835 exhibited the highest continuity with a cumulative size of 44.26 Mbp for the first ten scaffolds/contigs, surpassing the assemblies of all examined *Fp* strains. Our findings of the high-quality genome of *Fp* DSM106835 provide an important resource to investigate its genetics, biology and evolutionary history. This study also contributes to fulfill the gaps in fungal knowledge, particularly the genes/metabolites associated with pathogenicity during the plant-pathogen interaction responsible for SDS.

## Background & Summary

Date palm (*Phoenix dactylifera*) is considered as one of the most economically important fruit crops grown in arid lands of the Arabian Peninsula, the Middle East and North Africa. This evergreen tree is well-adapted to harsh desert conditions of long hot summers, little rainfall and low humidity. The United Arab Emirates (UAE) has the largest number of date palms in the world, and is considered among the top global exporters of dates^1^. On the other hand, date palm orchards in the UAE have recently been suffering from serious diseases caused by fungal pathogens^2,3^, including sudden death syndrome (SDS; also known as date palm wilt disease)^4^.

Although researchers have reported several *Fusarium* species that are associated with disease symptoms of SDS worldwide^3,5–7^, *Fusarium oxysporum* f.sp. *cumini* (*Foc*) DSM106834, *F. proliferatum* (*Fp*) DSM106835 and *F. solani* (*Fs*) DSM106836 are the causal agents of SDS on date palm in the UAE^4^. In North Africa, Bayoud is the most destructive fungal disease of date palm that is linked with *F. oxysporum* f.sp. *albedinis* (*Foa*)^8,9^. *Fs* was, however, found associated with declined date palm trees in Pakistan^10^. In the UAE, *Fp* was identified the main *Fusarium* spp. causing SDS in Saudi Arabia, Iraq, Jordan and Tunisia^11–14^.

The soil-borne filamentous fungus *Fp* is a plant pathogen that belongs to the family Nectiraceae from the division Ascomycota. *Fp* is part of the *F. fujikuroi* species complex (FFSC) that is composed of around 60 different phylogenetic species with phytopathological and clinical relevance^15,16^. As other *Fusarium* spp., *Fp* has the ability to produce the mycotoxin, fumonisin^17,18^. Fumonisins are carcinogenic, estrogenic and immune suppressive in mammals and may cause birth defects of the brain and spinal cord^18,19^. Other mycotoxins, such as beauvericin, enniatins and moniliformin, can also be produced by *Fp* and act as virulence factors and specific effectors to elicit resistance to SDS in date palm^11,13,14^.

Although SDS has been reported to negatively affect date palm plantations in the UAE and elsewhere, the genetic information of the causal agent is still meager. Therefore, we developed a whole genome sequencing of *Fp* DSM106835 using PacBio^®^ to provide high throughput sequencing with highly accurate long HiFi reads. Here, we presented a highly contiguous and complete *de novo* genome assembly for *Fp* DSM106835, the main causal agent of SDS on date palm in the UAE, using PacBio HiFi long-reads and Omni-C data. The final genome is about 58.5 Mbp across 418 scaffolds, with a scaffold N50 of 4.4 Mbp and a Benchmarking Universal Single-Copy Orthologs (BUSCO)^20^ score of 99.2%. This genome adds a valuable resource for studying the evolutionarily relationships and elucidating the molecular mechanisms for host specificity to further improve our understanding of *Fp* DSM106835-date palm interaction.

## Methods

### Growth and culture maintenance of *F. proliferatum* DSM106835

**Fig. 1:**
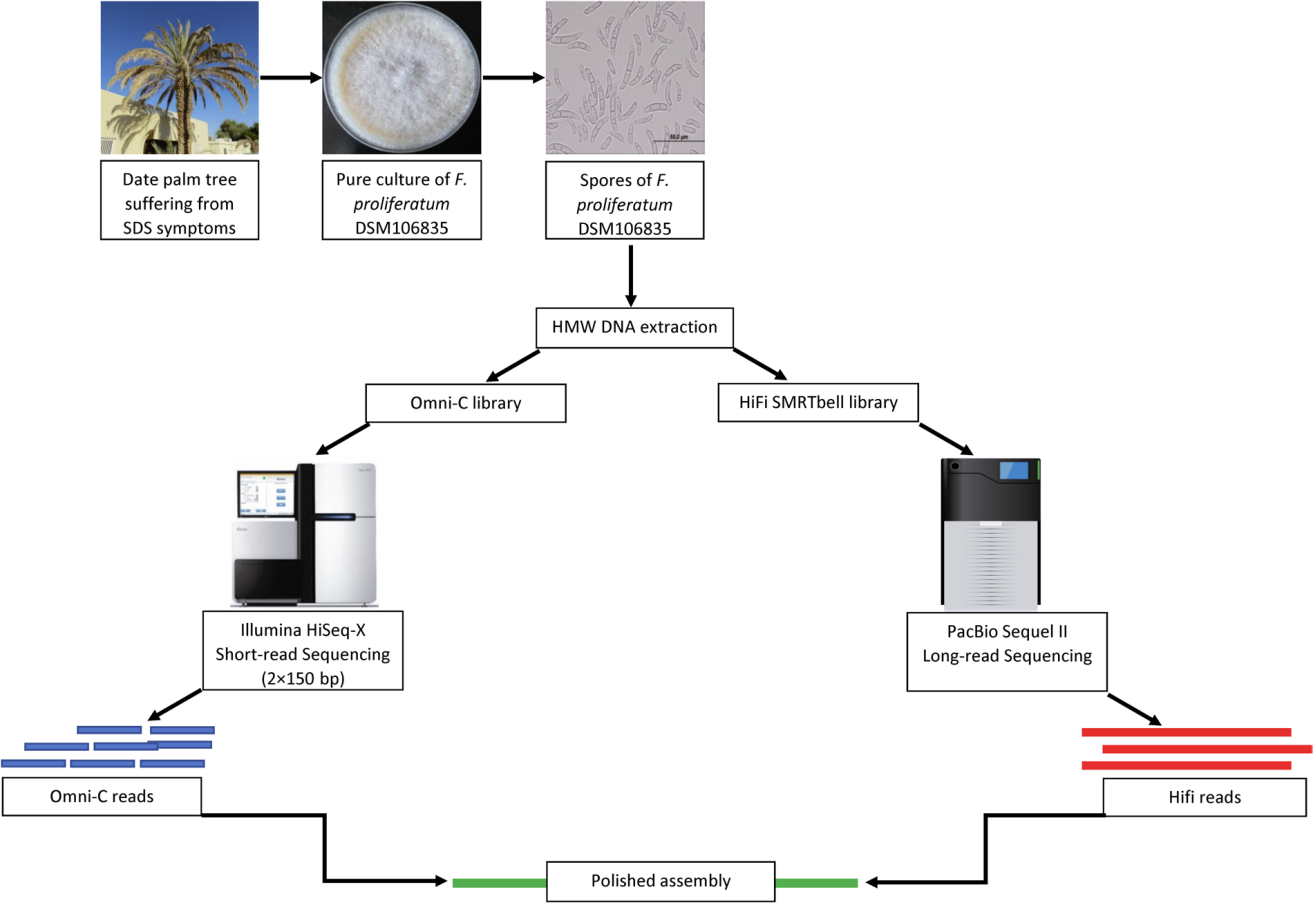
Flow diagram of the isolation, genome sequencing and assembly of *Fusarium proliferatum* DSM106835. Date palm trees showing symptoms of SDS were used to establish a pure culture of *F. proliferatum* DSM106835. Spores produced by the fungal pathogen were observed under light microscopy and further used for HMW DNA extraction. Omni-C and HiFi SMRbell libraries were prepared for Illumina HiSeq-X (short-read sequencing) and PacBio^®^ Sequel II (long-read sequencing), respectively. HiFi and Omni-C reads were merged to develop a long-read-only assembly where all chromosomes were present as single contigs without the introduction of artificial gaps (Courtesy of Illumina, Inc., Pacific Biosciences of California, Inc.). SDS, sudden death syndrome; HMW, high molecular weight.

The pathogen, *Fp* DSM106835, was previously isolated from date palm trees showing SDS symptoms from Al Wagan area in Al Ain, Abu Dhabi, UAE, grown and maintained in potato dextrose agar plates (PDA; Lab M Limited, Lancashire, UK) supplemented with 25 mg/L penicillin-streptomycin (Sigma-Aldrich Chemie GmbH, Taufkirchen, Germany) at 25°C^4^. Plates were subcultured every 14 days on PDA plates until pure *Fp* DSM106835 colonies were obtained. A flow scheme of the isolation and culturing of *Fp* DSM106835 can be found in Fig. 1.

### DNA extraction and PacBio HiFi sequencing

High molecular weight (HMW) DNA was extracted by first scraping all visible fungal material from the Petri dish, which was then transferred to a 50-ml tube with 2-ml of autoclaved ddH_2_O, flash frozen to create a pellet of ~500 mg, and ground to become powder. In the ground sample, 10 ml of cetyltrimethyl ammonium bromide (CTAB) and 100 µl of β-mercaptoethanol (BME) were added and incubated at 68°C for 15 minutes. After incubation, 10 µl of protease and 1 µl of RNase were added to the sample and incubated at 60°C for 30 minutes. Phenol/chloroform/isoamyl-alcohol was used to extract DNA from the cell lysate, which was then centrifuged into a pellet. The formed pellet was resuspended in 200 µl Tris-EDTA buffer (TE buffer). DNA samples were first sequenced using the PacBio Sequel II sequencer at Dovetail Genomics (Scotts Valley, California, USA). This sequencing step was carried out by preparing PacBio SMRTbell libraries (∼20 kbp) using the SMRTbell Express Template Prep Kit 2.0 (PacBio, Menlo Park, CA), according to the manufacturer’s protocol.

### Omni-C sequencing

Omni-C sequencing is a chromatin conformation capture technology that allows the investigation of the genome’s three-dimensional (3D) organisation. The Omni-C library was prepared using the Dovetail^®^ Omni-C^®^ Kit according to the manufacturer’s protocol. Briefly, the chromatin was fixed with disuccinimidyl glutarate (DSG) and formaldehyde in the nucleus. The crosslinked chromatin was *in situ* digested with DNaseI.

After digestion, chromatin fragments attached to Chromatin Capture Beads were released by lysing the cells with sodium dodecyl sulfate (SDS) buffer. The chromatin ends were repaired followed by ligation to a biotinylated bridge adapter. After proximity ligation, crosslinks were reversed and DNA was purified. The sequencing librararies using Illumina-compatible adaptors were generated. Biotin-containing fragments were isolated using streptavidin beads before PCR amplification. The library was sequenced on an Illumina HiSeq-X platform. A flow scheme of HMW DNA extraction, library preparations and genome assembly of *Fp* DSM106835 can be found in Fig. 1.

### *De novo* genome assembly

**Fig. 2:**
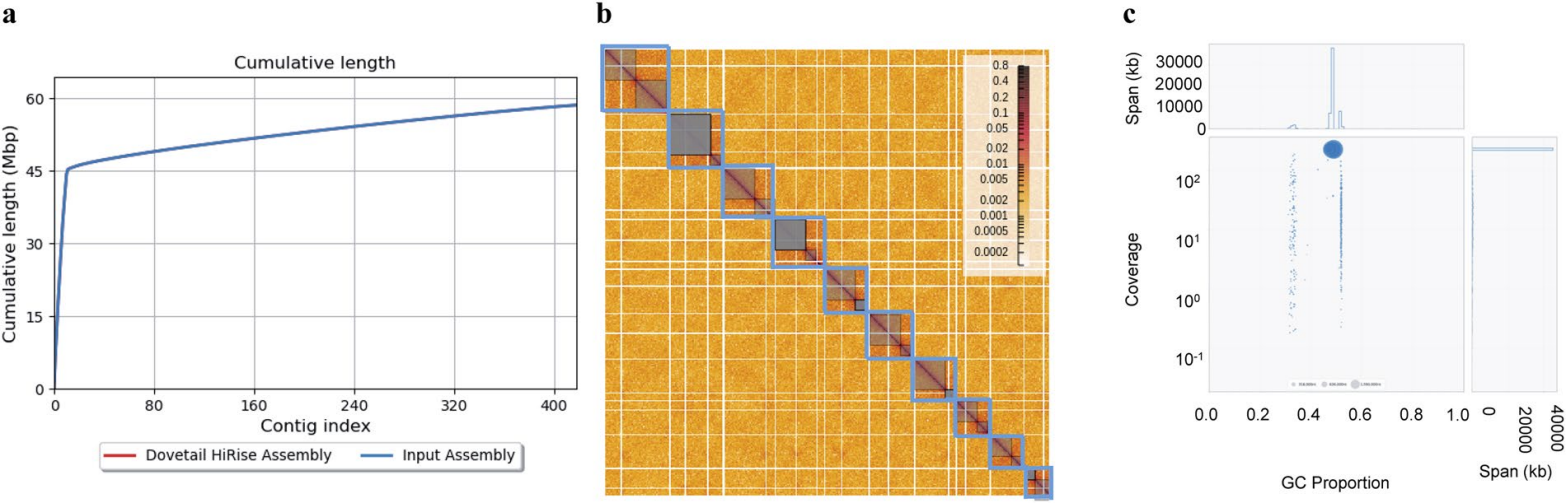
Taxonomic partitioning, average read length of the raw data and Omni-C contact map of *Fusarium proliferatum* DSM106835. (**a**) The Cumulative length of scaffolds for the assembly; (**b**) Omni-C contact map showing the intensity of the physical interaction between genome regions; and (**c**) Taxonomic partitioning of *F. proliferatum* DSM106835 raw reads generated using blobtools2. In (**b**), the primary 10 chromosome-length scaffolds are highlighted in blue. In (**c**), blue represents Ascomycota while grey represents the reads with no-hits.

The genome assembly was carried out by first using 26.9 Gbp of PacBio Circular Consensus Sequencing (CCS) reads as an input to the hifiasm assembler^21^ with default parameters to create the initial *de novo* assembly. Omni-C sequencing resulted in a paired-end set of raw reads, each 11,489,515 bp in length and GC content of 49% (Table 1). These reads, along with the *de novo* assembly, were used as input data for HiRise^22^, a software pipeline explicitly designed for using proximity ligation data to scaffold genome assemblies (Fig. 2a). Dovetail Omni-C library sequences were aligned to the draft input assembly using BWA^23^, and pairtools^24^ was used to remove the PCR duplicates from the assembly; followed by SAMtools^25^ to generate the final bam file. Quality control using the script get_qc.py part of the HiRise package found 88,132,543 (76.71%) of read pairs were mapped and 12,232,575 (10.65%) were unmapped. The HiRise pipeline was used to identify misassemblies, and to break and sort scaffolds (only those above the threshold) in accordance with the likelihood model used by HiRise. Omni-C contact maps were created from the output of HiRise using Juicer^26^, and the contact map was configured to identify Topologically Associated Domains and A/B genome compartments. The configured contact map was visualised using Juicebox^27^ (Fig. 2b). The final *de novo* assembly of 58,468,907 bp in length had an N50 value of 4,383,091. This assembly was used as a query to perform a BLASTN^28^ search against the National Center for Biotechnology Information (NCBI) nucleotide database^29^ as an input for blobtools2^30^ to visualise the assembly and its taxonomic partitioning (Fig. 2c). The HiCanu^31^ assembler was also used to assemble the genome to compare and validate the hifiasm assembly. The completeness of the final assembly was assessed using BUSCO with fungi_odb10 lineage-specific profile^32^.

**Table 1:** Information on the assembly of *Fusarium proliferatum* DSM106835.

Reads	Total HiFi CCS reads	1,754,151
	Total HiFi CCS read size (bp)	26,392,037,220
	Average coverage (X)	503
	Mean read size (bp)	15,045
	Median read size (bp)	14,761
	N50 read size (bp)	15,154
	Max read size (bp)	49,366
	Total Omni-C reads	114,895,105
	Total Omni-C read size (bp)	17,234,265,750
Assembly	Genome size (bp)	58,468,907
	Estimated genome size by K-mer analysis (Mbp)	47.07
	No. of scaffolds	418
	Scaffold N50	4,383,091

### Transposable element analysis, gene prediction and annotation

The assembly of *Fp* DSM106835 was subjected to transposable element (TE) analysis using a customised repeat annotation pipeline. This pipeline incorporated multiple *de novo* TE discovery tools, including RepeatModeler^33^, HelitronScanner^34^, MITE Tracker^35^, SINEScan^36^, and RepeatMasker. In brief, RepeatModeler integrates RECON^37^, RepeatScout^38^, and LTRHavest/LTRretriver^39^. These tools obtained a comprehensive representation of TEs, leading to a relatively complete TE library. Subsequently, RepeatMasker was employed with this library to identify genome-wide TEs and mask all the repeats and tandem sequences. The resulting masked genome sequences were then subjected to *de novo* gene prediction and annotation using BRAKER 2^40^. In the BRAKER 2 pipeline, Augustus^41^ was trained with protein sequences of orthologous genes in fungi genomes to help in gene prediction. The genome was then subjected to functional annotation and Gene Ontology (GO) analysis using Blast2GO^42^, and the prediction of secondary metabolites was performed using fungal-antiSMASH^43^.

### Assessment of completeness and continuity of the genome assembly

For assembly continuity comparison, the genome sequences of seven *Fp* strains with gene annotations, ET1 (FJOF00000000)^44^, FFSC RH7 (JAJALB000000000)^45^, Fp_A8 (MRDB00000000)^46^, ITEM2341 (PKMI00000000)^47^, MPVP328 (PKMJ00000000)^48^, NRRL62905 (FCQG00000000)^49^, and R16 (PKMG00000000)^50^ were downloaded from the NCBI database. These strains were compared against *Fp* DSM106835 by comparing the sequence length of each assembly with the average scaffold length, and completeness analysis was performed by comparing the results of BUSCO analysis of each genome against fungi_odb10 lineage-specific profile.

## Data Records

All sequence data, including raw HiFi long reads and Omni-C short reads, were deposited to the NCBI database under BioProject PRJEB64160, with accessions ERR11733479^51^ and ERR11733478^52^, respectively. The genome assembly is available through NCBI GenBank with the accession CAUHTQ000000000^53^. The genome annotation information was deposited in the Figshare database^54^.

## Technical Validation

### Evaluating the quality of the genome assembly

The PacBio sequencing produced 1,754,151 raw HiFi long reads with an average read length of 15,045.5 bp, resulting in 26.4 Gbp, mostly falling between 5,000–25,000 bp in length and approximately 560x coverage (Supplementary Fig. S1). By utilising the hifiasm and HiRise software, the assembly of HiFi reads with Omni-C reads generated 418 scaffolds, amounting to 58.47 Mbp. The N50 value was 4.38 Mbp. The largest 11 scaffolds had a combined size of 45.18 Mbp, which accounted for 77.3% of the entire genome (Table 1). Similar results were obtained when the assembly of HiCanu was compared to that using hifiasm (Supplementary Fig. S2). The assembly achieved a completeness rate of 99.2% for the 758 orthologous genes in fungi_odb10 using BUSCO, similar to the genome assembly of *Fp* strain Fp_A8 (99.3%; Table 1).

### Genome annotation

**Fig. 3:**
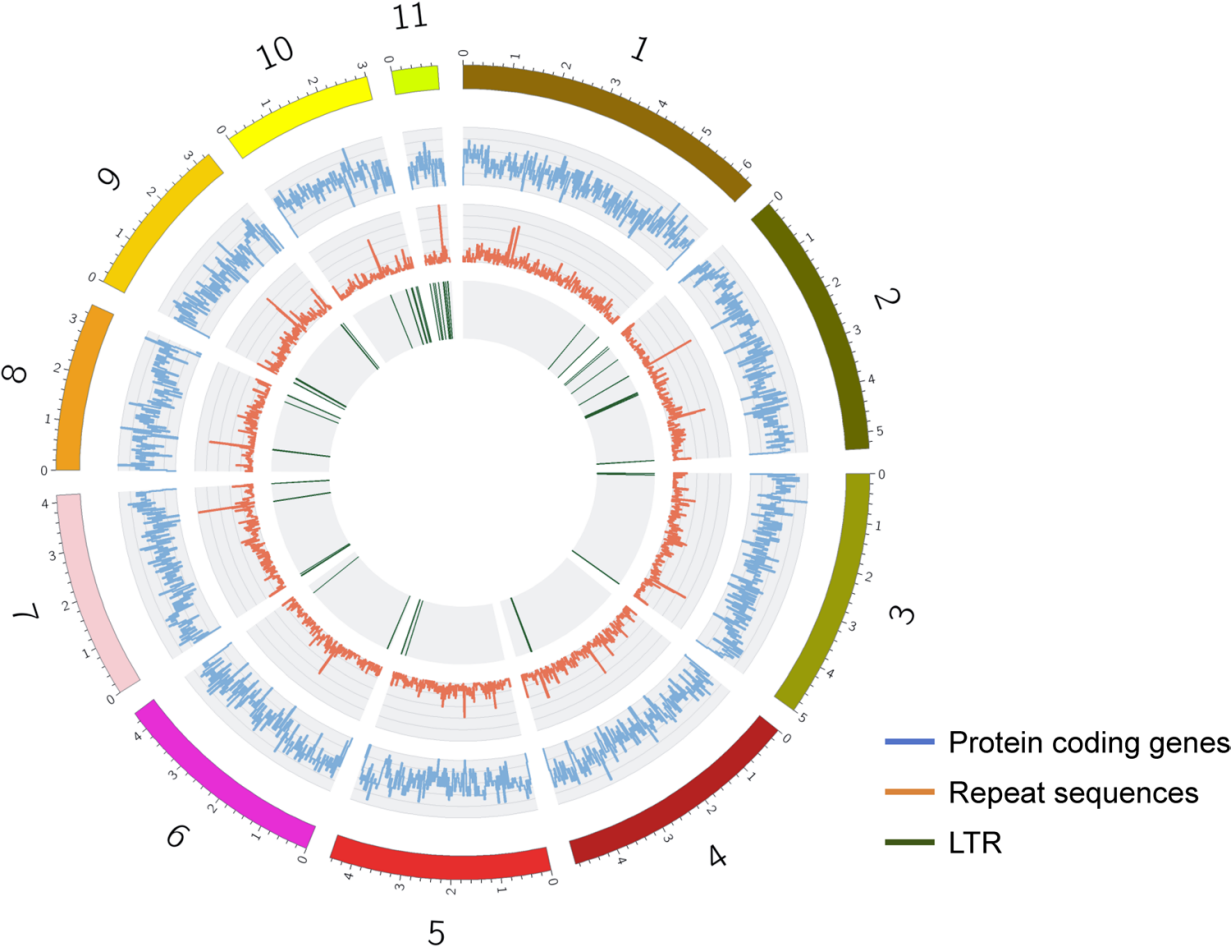
Circos map of the 11 significant scaffolds for *Fusarium proliferatum* DSM106835. Outer track represents the ideogram of 11 scaffolds. The bin size of each track was 20 Kbp. LTR, long terminal repeats.

**Fig. 4:**
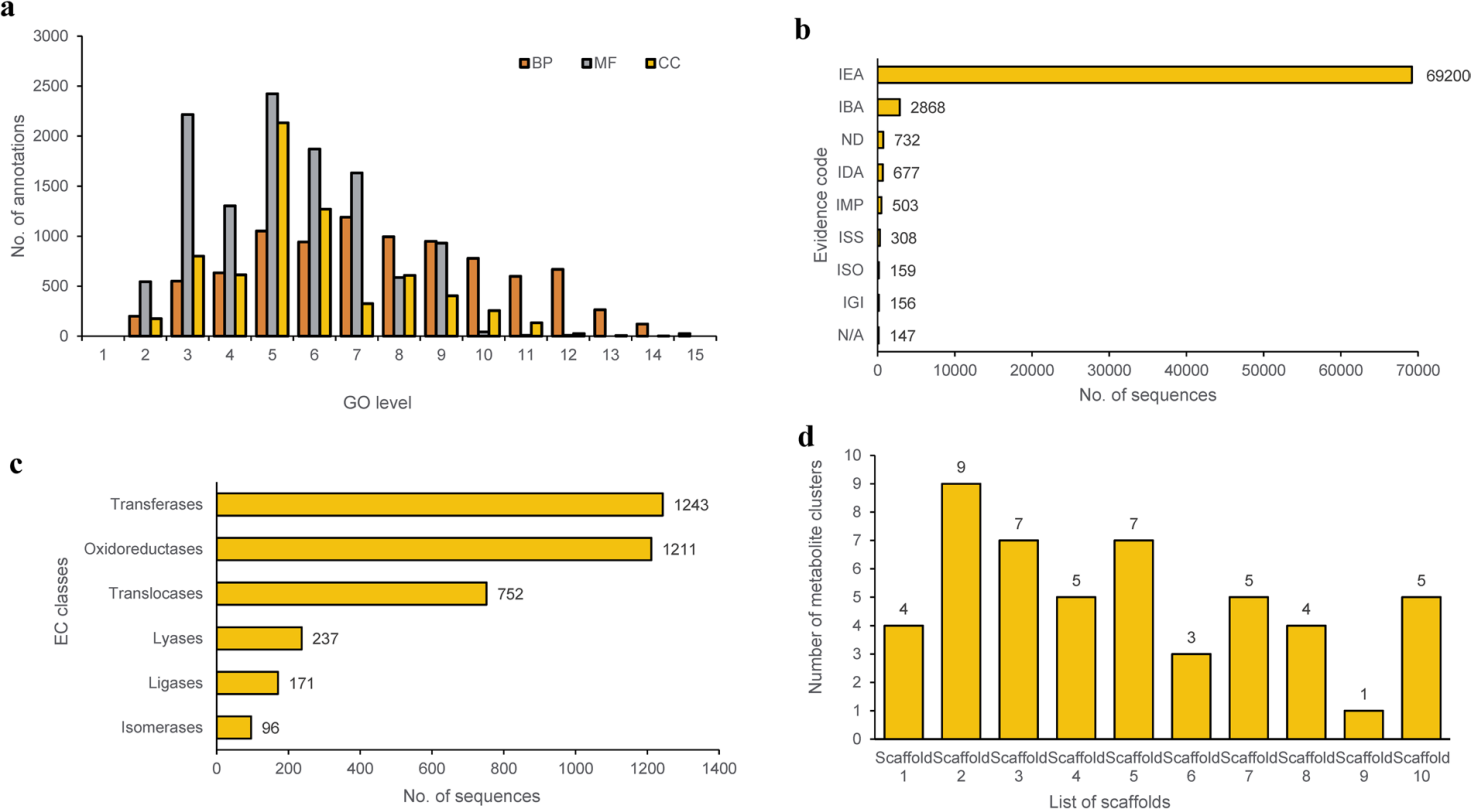
Functional annotation and Gene Ontology distribution for *Fusarium proliferatum* DSM106835. (**a**) Distribution of GO generated from the genome of *F. proliferatum* DSM106835; and (**b**) evidence code distribution for the obtained sequences. (**c**) EC classification for sequences present in the assembly; and (**d**) the number of secondary metabolite biosynthesis gene clusters identified from the first 11 scaffolds of the genome of *F. proliferatum* DSM106835. In (**b**), the distribution of evidence code for functional terms was obtained during the mapping step. GO, Gene Ontology; BP, biological process; MF, molecular function; CC, cellular component; EC, enzyme code.

A total of 3.96 Mbp of transposable repeat sequences were detected in the genome of *Fp* DSM106835, including retroelements (0.48 Mbp), DNA transposons (0.39 Mbp), rolling-circle replicates (Helitrons; 1.52 Mbp), and some unclassified repeats (1.56 Mbp), collectively constituting 6.76% of the total genome (Table 2; Fig. 3). Notably, the genome of *Fp* DSM106835 also included long terminal repeat (LTR) retroelements that belong to Gypsy superfamily. Heitron rolling-circle elements and unclassified elements accounted for a significant part of repeat sequences. The gene prediction using BRAKER2^45^ resulted in 15,580 putative genes, of which 267 were TE and 15,313 were non-TE genes. We also detected 16,321 transcripts, where the average gene length was about 1,580 bp. After performing functional annotation on the predicted sequences, GO terms distribution for cellular components, molecular function, and biological processes was identified (Fig. 4a) with the highest number of annotations belonging to GO levels 3–7. The evidence code distribution was calculated, and mostly they received a hit from Inferred from Electronic Annotation (IEA) and Inferred from Biological aspect of Ancestor (IBA) sections (Fig. 4b). Similarly, the enzyme code (EC) classification was carried out, from which most of the sequences were found to be either transferases or oxidoreductases (Fig. 4c).

**Table 2:** Repeat sequence analysis of the genome of *Fusarium proliferatum* DSM106835.

Type of Element	Number of Element	Length (bp)	Percentage of Sequence (%)
Retroelements	248	482,156	0.82
LINEs	66	215,895	0.37
LTR elements	182	266,261	0.46
Gypsy/DIRS1	46	222,508	0.38
DNA transposons	339	391,106	0.67
Tc1-IS630-Pogo	269	161,039	0.28
Rolling-circles	3239	1,522,651	2.60
Unclassified	1610	1,560,876	2.67
Small RNA	2490	2,632,552	4.50
Simple repeats	6204	248,208	0.42
Low complexity	649	31,602	0.05
Total	15342	7,734,854	13.23

The number of secondary metabolite biosynthesis gene clusters was also identified (Fig. 4d). In general, various gene clusters ranging from clinically relevant fumonisins, virulence-related ACT-Toxin II, and phytotoxic destruxin A were present in the genome. Gene clusters of secondary metabolites were found to belong to the biosynthesis of fusaric acid, oxyjavanicin, gibberellin, bikaverin, ACT-Toxin II, koraiol, Fujikurin A, α-acorenol, NG-391 and Gibepyrone A (Table 3).

**Table 3:** List of secondary metabolite biosynthetic gene clusters identified from the genome of *Fusarium proliferatum* DSM106835 using antiSMASH.

Region	Type	From	To	Cluster similarity	Similarity (%)
2.1	T1PKS	81,001	185,855	asperfuranone	18%
2.3	T1PKS,NRPS	275,348	382,994	gibepyrone-A	100%
2.7	NRPS,T1PKS	2,708,378	2,756,301	NG-391	83%
2.8	NRPS	4,987,936	5,037,349	beauvericin	20%
2.9	T1PKS	5,121,665	5,169,546	fumonisin	52%
3.6	NRPS,T1PKS	4,661,484	4,713,770	equisetin	54%
3.7	NRPS-like,T1PKS	4,897,251	4,974,345	fusaric acid	72%
4.3	terpene	3,396,575	3,426,909	squalestatin S1	40%
4.5	T1PKS	4,082,972	4,130,027	oxyjavanicin	100%
5.2	NRPS-like,NRPS	94,150	150,568	destruxin A	9%
5.3	terpene	205,874	230,579	gibberellin	100%
5.6	T1PKS	4,268,976	4,313,388	bikaverin	71%
5.7	NRPS-like	4,318,228	4,372,942	fusaridione A	12%
7.5	NRPS	4,056,001	4,098,456	acetylaranotin	40%
8.1	NRPS,T1PKS	620,775	672,633	ACT-Toxin II	100%
8.2	terpene	940,349	985,876	koraiol	100%
8.3	T1PKS	1,882,799	1,926,993	fujikurin A/B/C/D	100%
8.4	T1PKS	2,802,293	2,844,647	solanapyrone D	33%
10.1	T1PKS	396,309	444,038	neurosporin A	20%
10.4	terpene	2,119,715	2,146,446	α-acorenol	100%

### Genome continuity and completeness analysis

**Fig. 5:**
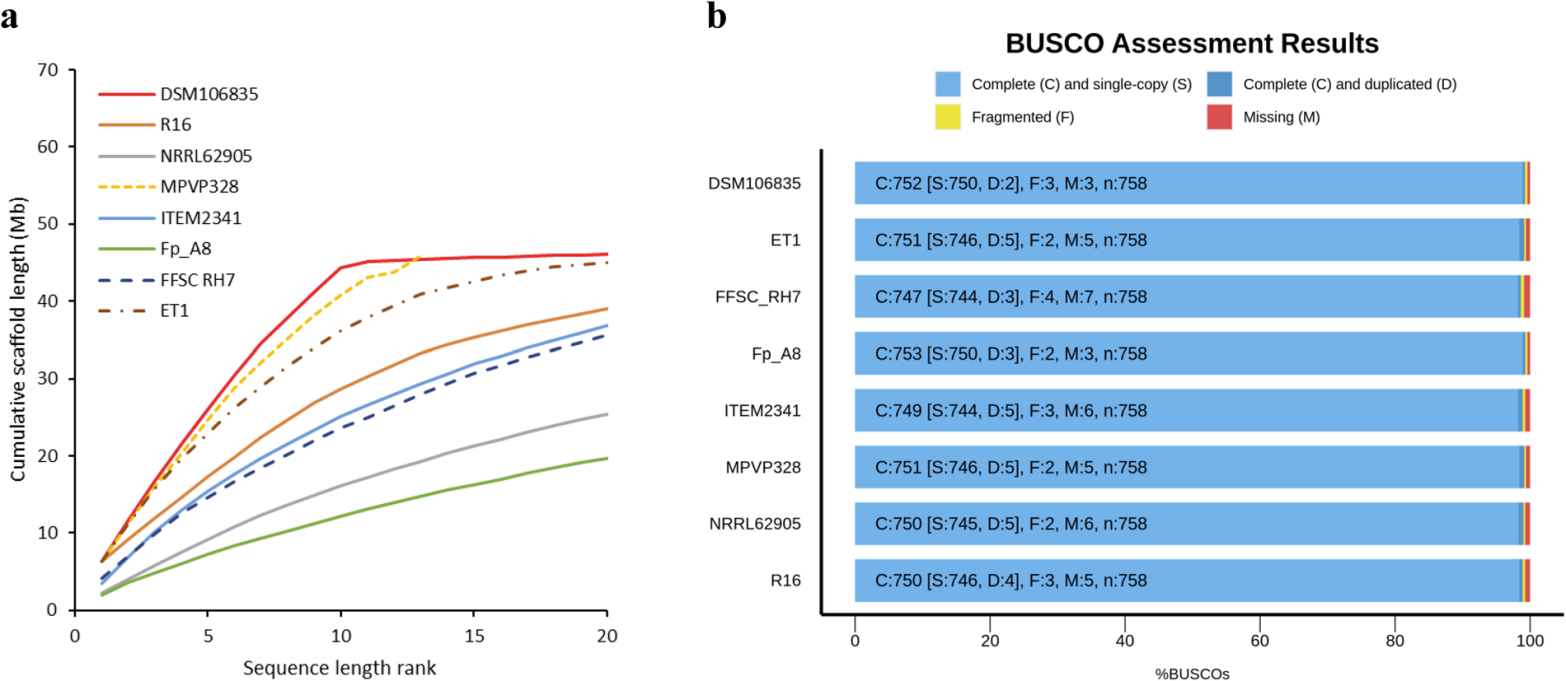
Contiguity and completeness of the assembly of *Fusarium proliferatum* DSM106835. (**a**) Contiguity; and (**b**) completeness of the assembly of *F. proliferatum* DSM106835 compared with assemblies of seven *F. proliferatum* strains. In (**a**), only the first 20 longest scaffolds were presented.

The continuity analysis revealed that *Fp* DSM106835 exhibited the highest continuity among the seven *Fp* strains collected from NCBI. The cumulative size of the first 10 scaffolds/contigs was 44.26 Mbp, which surpassed the assemblies of all other *Fp* strains ranging from 12.19 Mbp in *Fp* Fp_A8) to 36.19 Mbp in *Fp* ET1 (Fig. 5a). The same genomes were compared for their completeness using BUSCO^19^, and *Fp* DSM106835 achieved a completeness rate of 99.2% for the 758 orthologous genes in the Fungi_odb10 database, which is comparable to *Fp* Fp_A8 (99.3%; Fig. 5b).

### Supplementary information

Supplementary Information

## Data Availability

This work did not utilise a custom script. Data processing was carried out using the protocols and manuals of the relevant bioinformatics software.
