# Supplementary material for: Fully resolved assembly of Fusarium proliferatum DSM106835 genome
Source: Sci Data. 2023 Oct 16;10:705. doi: 10.1038/s41597-023-02610-4 (PMC10579329; doi:10.1038/s41597-023-02610-4)
Supplement: Supplementary file 1 — Supplementary Information [file 41597_2023_2610_MOESM1_ESM.pdf]

Fully resolved assembly of *Fusarium proliferatum* DSM106835 genome

Authors

Gouthaman P. Purayil[1], Amal A. Almarzooqi[1], Khaled A. El-Tarabily[1,*], Frank M. You[2*] and Synan F. AbuQamar[1*]

Affiliations

[1] Department of Biology, College of Science, United Arab Emirates University, Al Ain, 15551, United Arab Emirates
[2] Ottawa Research and Development Centre, Agriculture and Agri-Food Canada, 960 Carling Avenue, Ottawa, ON K1A 0C6, Canada

∗Corresponding authors: Khaled A. El-Tarabily, E-mail: ktarabily@uaeu.ac.ae; Frank M. You, E-mail: frank.you@agr.gc.ca; Synan F. AbuQamar, E-mail: sabuqamar@uaeu.ac.ac

Supplementary Material

Supplementary Fig. S1
PacBio sequencing of *Fusarium proliferatum* DSM106835. The sequencing produced 1,754,151 raw HiFi reads and resulted in 26.4 Gbp. (A) Most reads fall between 5000-25000 bp length; and (B) approximately 560x coverage based on k-mer distribution.

Supplementary Fig. S2
Comparison of genome assemblies of *Fusarium proliferatum* DSM106835 between Hifiasm and HiCanu. Alignment of genome of assemblies of *F. proliferatum* DSM106835 using HiCanu (x-axis) against Hifiasm (y-axis) to measure the percentage of identity between the genomes and validate the similarity in the final assemblies produced using different assembly tools.

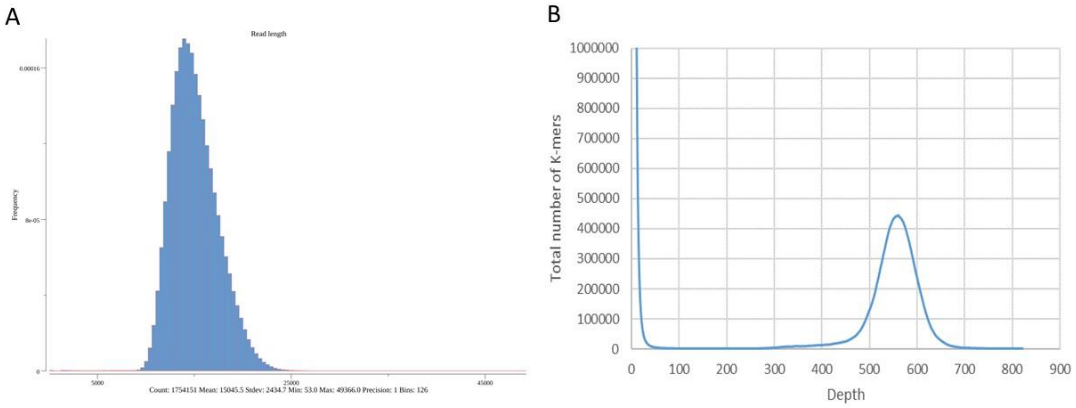

Supplementary Fig. S1

PacBio sequencing produced 1,754,151 raw HiFi reads, resulting in 26.4 Gbp. (A) Most reads fall between 5000-25000 bp length, and (B) approximately 560x coverage based on k-mer distribution.

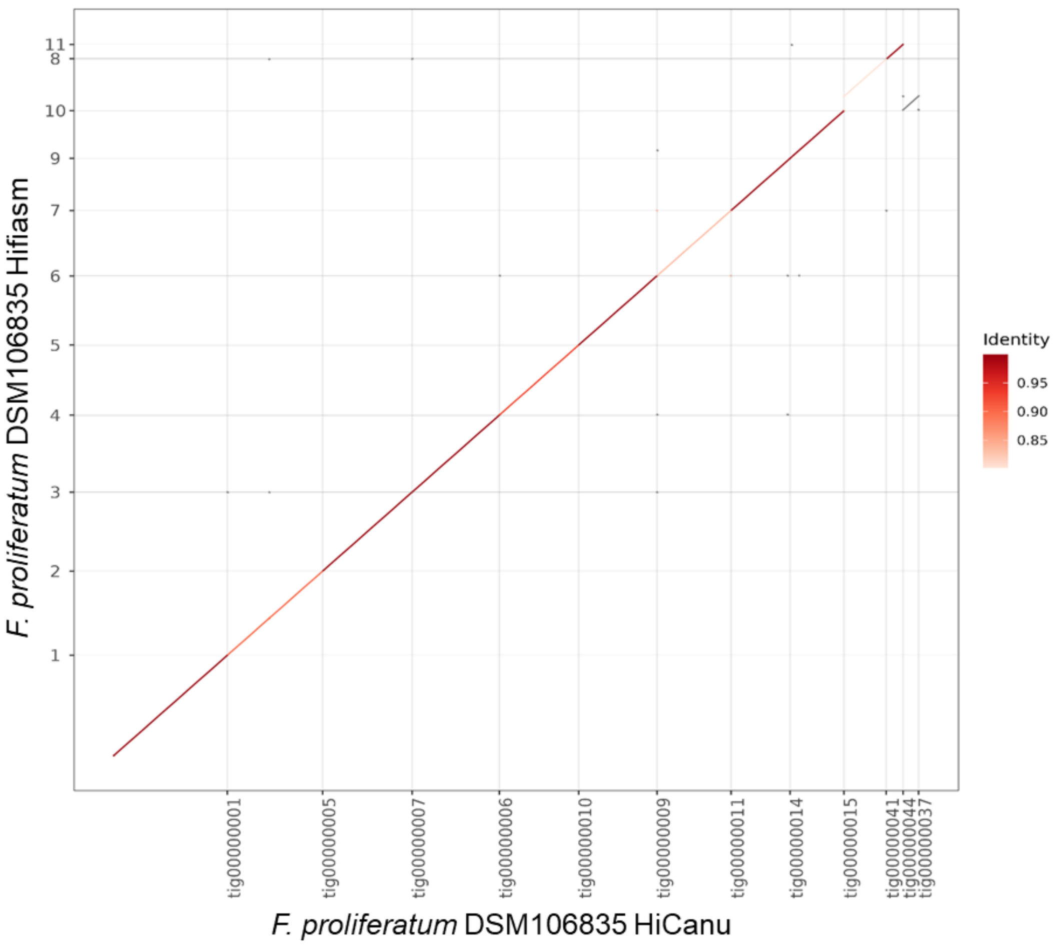

Supplementary Fig. S2

Comparison of genome assemblies of *Fusarium proliferatum* DSM106835 between Hifiasm and HiCanu. Alignment of genome of assemblies of *F. proliferatum* DSM106835 using HiCanu (x-axis) against Hifiasm (y-axis) to measure the percentage of identity between the genomes and validate the similarity in the final assemblies produced using different assembly tools.
